# For the road: calibrated maternal investment in light of extracellular symbiont transmission

**DOI:** 10.1098/rspb.2022.0386

**Published:** 2022-04-27

**Authors:** Inès Pons, Miguel Ángel González Porras, Noa Breitenbach, Jürgen Berger, Katharina Hipp, Hassan Salem

**Affiliations:** ^1^ Mutualisms Research Group, Max Planck Institute for Biology, Tübingen 72076, Germany; ^2^ Electron Microscopy Facility, Max Planck Institute for Biology, Tübingen 72076, Germany

**Keywords:** symbiosis, symbiont transmission, maternal investment, host–microbe coevolution

## Abstract

Faithful transmission of beneficial symbionts is critical for the persistence of mutualisms. Many insect groups rely on extracellular routes that require microbial symbionts to survive outside the host during transfer. However, given a prolonged aposymbiotic phase in offspring, how do mothers mitigate the risk of symbiont loss due to unsuccessful transmission? Here, we investigated symbiont regulation and reacquisition during extracellular transfer in the tortoise beetle, *Chelymorpha alternans* (Coleoptera: Cassidinae). Like many cassidines, *C. alternans* relies on egg caplets to vertically propagate its obligate symbiont *Candidatus* Stammera capleta. On average, each caplet is supplied with 12 symbiont-bearing spheres where *Stammera* is embedded. We observe limited deviation (±2.3) in the number of spheres allocated to each caplet, indicating strict maternal control over symbiont supply. Larvae acquire *Stammera* 1 day prior to eclosion but are unable to do so after hatching, suggesting that a specific developmental window governs symbiont uptake. Experimentally manipulating the number of spheres available to each egg revealed that a single sphere is sufficient to ensure successful colonization by *Stammera* relative to the 12 typically packaged within a caplet. Collectively, our findings shed light on a tightly regulated symbiont transmission cycle optimized to ensure extracellular transfer.

## 1. Introduction

Numerous adaptations in insects reflect a symbiotic condition. By deriving essential nutrients to exploit an imbalanced diet [1–5], or defensive compounds to fend off parasitic and pathogenic threats [6–8], many insect clades obligately rely on mutualistic microbes for development, reproduction and survival [9–13]. Correspondingly, insects evolved a diversity of structures and behaviours to ensure the persistence of these partnerships across generations [14–16].

Vertical transmission of beneficial microbes is a common characteristic of obligate symbioses [15–17]. Mechanisms ensuring transfer contribute to the stability of mutualisms by reducing the risk of symbiont loss, while mitigating the exposure of juvenile stages to pathogens and parasites [18]. Intracellular symbionts inhabiting bacteriocytes are typically transferred transovarially during the early stages of oogenesis or embryogenesis [19–21]. Alternatively, numerous insect groups rely on extracellular routes to vertically propagate their symbionts. From egg smearing [22,23] to microbe-embedding secretions [24–26], these routes are unified by the symbiont's ability to survive outside the host. Despite the prevalence of extracellular symbionts in insects and their demonstrated functional importance [14], mechanistic insights into how these microbes are regulated, allocated and, ultimately, reacquired during transfer is largely unexplored for most study systems (but see [27–30]). We pursue these aims in tortoise beetles (Coleoptera: Chrysomelidea: Cassidinae), given their obligate symbiosis with *Candidatus* Stammera capleta.

Cassidines harbour *Stammera* in foregut symbiotic organs, in addition to ovary-associated glands in females [31,32]. Despite its extracellular localization throughout host development, *Stammera* possesses a drastically reduced genome (0.2 Mb) with a high AT composition [31,33,34]. The symbiont's streamlined metabolism is largely dedicated to informational processing (replication, transcription and translation) and the production of pectin-degrading enzymes [31,33,34]. Pectinases supplemented by *Stammera* are critical in allowing cassidines to digest plant leaves rich in recalcitrant pectins, such as homogalacturonan and rhamnogalacturonan I [35]. Symbiont loss results in a diminished pectinolytic phenotype and low larval survivorship [31], reflecting the key role *Stammera* plays in the digestive physiology of its beetle host [33].

**Figure 1.**
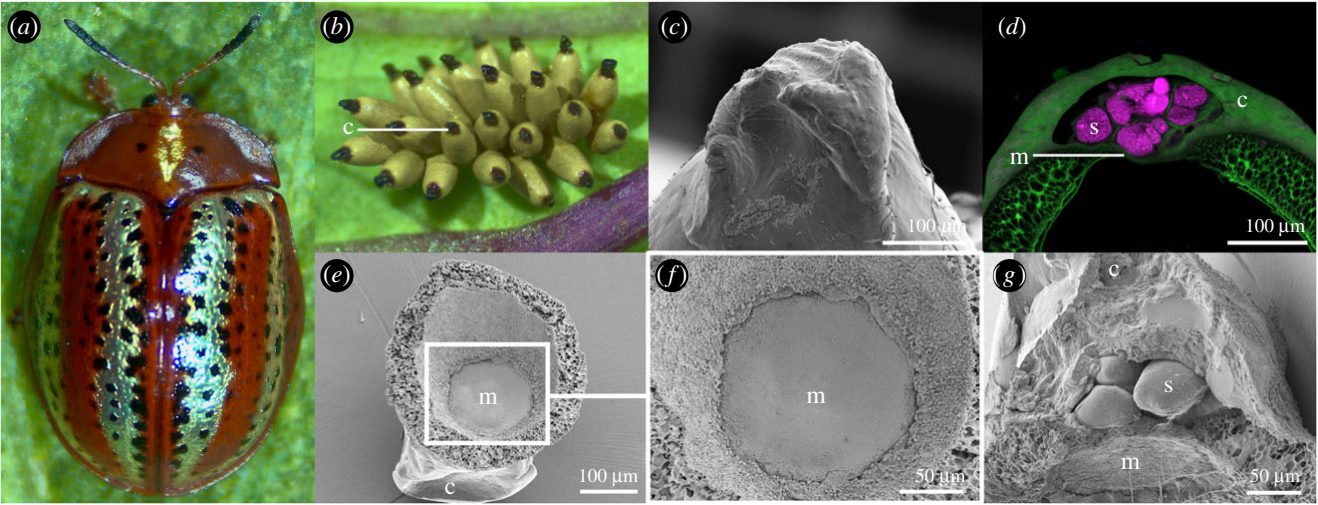
Structure and content of symbiont-bearing egg caplets. (*a*) The tortoise beetle, *Chelymorpha alternans*. (*b*) Eggs deposited on the underside of an *Ipomoea batatas* leaf, each topped with a caplet at the anterior pole. (*c*) Scanning electron microscopy (SEM) image outlining the exterior features of the egg caplet. (*d*) Fluorescence *in situ* hybridization (FISH) cross-section of a caplet (green: autofluorescence) and its enclosed spheres where *Stammera* (magenta: 16 rRNA probe) is embedded. (*e,f*) SEM images of an inverted caplet detached from the egg. Viewing direction is tilted by approximately 90°. (*g*) SEM cross-section of the egg caplet. Abbreviations: c, caplet; m, membrane; s, sphere. Scale bars are included for reference. (Online version in colour.)

In ensuring that future generations of cassidines are endowed with *Stammera*, females deposit symbiont-bearing ‘caplets’ at the anterior pole of each egg during oviposition [31]. Caplets are populated by spherical secretions where *Stammera* is embedded (figure 1*a–d*). While the consumption of *Stammera*-bearing spheres is predicted to initiate infection in cassidines [31], the timing of symbiont acquisition relative to larval hatching from the egg (eclosion) remains elusive. Since *Stammera* subsists extracellularly in the caplet while the embryo develops in the egg, mothers must balance symbiont allocation against the risk of aposymbiosis due to unsuccessful transmission. Cassidinae eggs hatch 7–14 days after oviposition [36,37], exposing symbiont-bearing caplets to several environmental stresses (e.g. high temperature, ultraviolet radiation and desiccation) that may mitigate symbiont viability within these structures.

In this study, using the cassidine *Chelymorpha alternans* as a model (figure 1*a*), we (i) determine the mechanism and timing of symbiont acquisition from the egg caplet, (ii) quantify within-caplet symbiont population dynamics during embryo development and eclosion from the egg, and (iii), through experimental manipulation, estimate the threshold number of *Stammera*-bearing spheres required for successful vertical transmission relative to the maternal endowment. Collectively, our findings shed light on a tightly regulated symbiont transmission cycle defined by a specific developmental window and calibrated host investment to ensure successful transfer.

## 2. Results and discussion

### (a) A membrane separates *Stammera* from the developing embryo in the egg

We applied confocal and scanning electron microscopy (SEM) to better characterize the content, structure and attachment of symbiont-bearing caplets to *C. alternans* eggs. Positioned at the anterior pole of each egg (figure 1*b,c*), the caplet and the spheres it encloses are separated from the developing embryo by a thin membrane (figure 1*d–g*). This may ensure that *Stammera* is only acquired when the gut, and its associated symbiotic organs, are fully formed during the secondary stages of embryo development. On average, each caplet encloses 12.45 (± 2.3) spheres where *Stammera* is embedded. The limited deviation observed between caplets relative to their associated spheres indicates that females strictly control how these symbiont-bearing secretions are allocated to each egg during oviposition, mirroring other highly selective vertical transmission strategies where symbiont supply is tightly calibrated during transfer [19,28,38]. Plataspid stinkbugs, which rely on egg capsules to vertically transmit their nutritional symbiont *Ishikawaella* display similar control over the microbe's allocation by depositing a single capsule for every four eggs irrespective of clutch size [28].

### (b) *Stammera* population is stable in the egg caplet and is acquired prior to hatching

**Figure 2.**
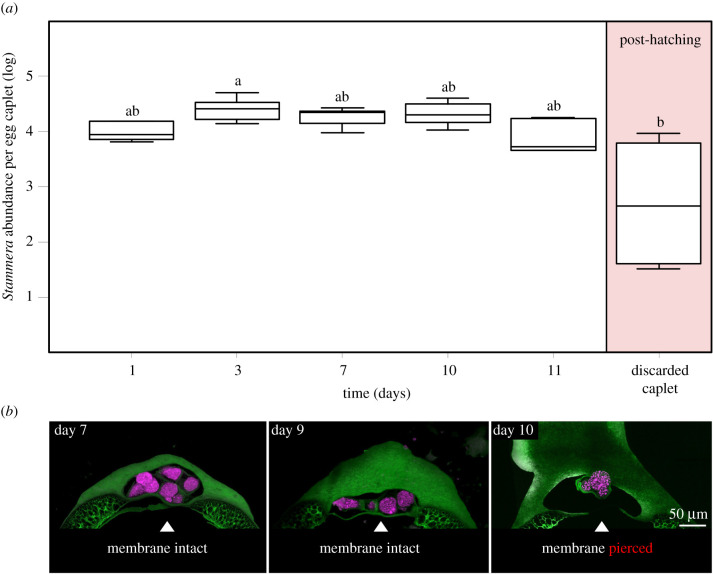
(*a*) Symbiont population dynamics in the caplet during embryo development in the egg following the quantification of 16S rRNA gene copy numbers. Lines represent medians, boxes indicate 25–75 percentiles, and whiskers denote range. Different letters above boxes indicate significant differences (LM, *p* = 0.016). (*b*) FISH cross-sections of egg caplets (green: autofluorescence) and its enclosed spheres where *Stammera* (magenta: 16 rRNA probe) is embedded 7, 9 and 10 days following oviposition. Membrane is intact up until day 10. Scale bar is included for reference. (Online version in colour.)

As *C. alternans* requires up to 11 days to complete its development in the egg prior to hatching [36], we aimed to describe *Stammera*'s population dynamics during that period. This revealed a symbiont population that is highly stable up until larvae eclose (figure 2*a*) (linear model (LM), *F*_5,23_ = 3.57, *p* = 0.016). While the membrane separating symbiont-bearing spheres from the embryo remains intact throughout development, it is pierced 1 day prior to hatching (figure 2*b*). This indicates that *C. alternans* does access its symbiont population while still in the egg, in contrast with other insect clades relying on extracellular symbiont transmission routes [14], such as egg smearing [22,23], and capsule- [25] and jelly-transmission [24]. These routes all require behavioural and structural adaptations facilitating post-hatch acquisition of beneficial microbes [14], usually through the active probing of symbiont-bearing eggshells [22], soil [39] or maternally provisioned secretions [24–27]. In quantifying symbiont abundance in caplets after hatching, and following larval abandonment of the egg clutch, we observe that more than a third (37%) of the symbiont's population is discarded along with the chorion (figure 2*a*) (LM, *F*_5,23_ = 3.57, *p* = 0.016), suggesting that maternal allocation of *Stammera* may greatly exceed the threshold necessary to ensure successful vertical transmission.

### (c) Aposymbiotic larvae do not reacquire *Stammera* after hatching

**Figure 3.**
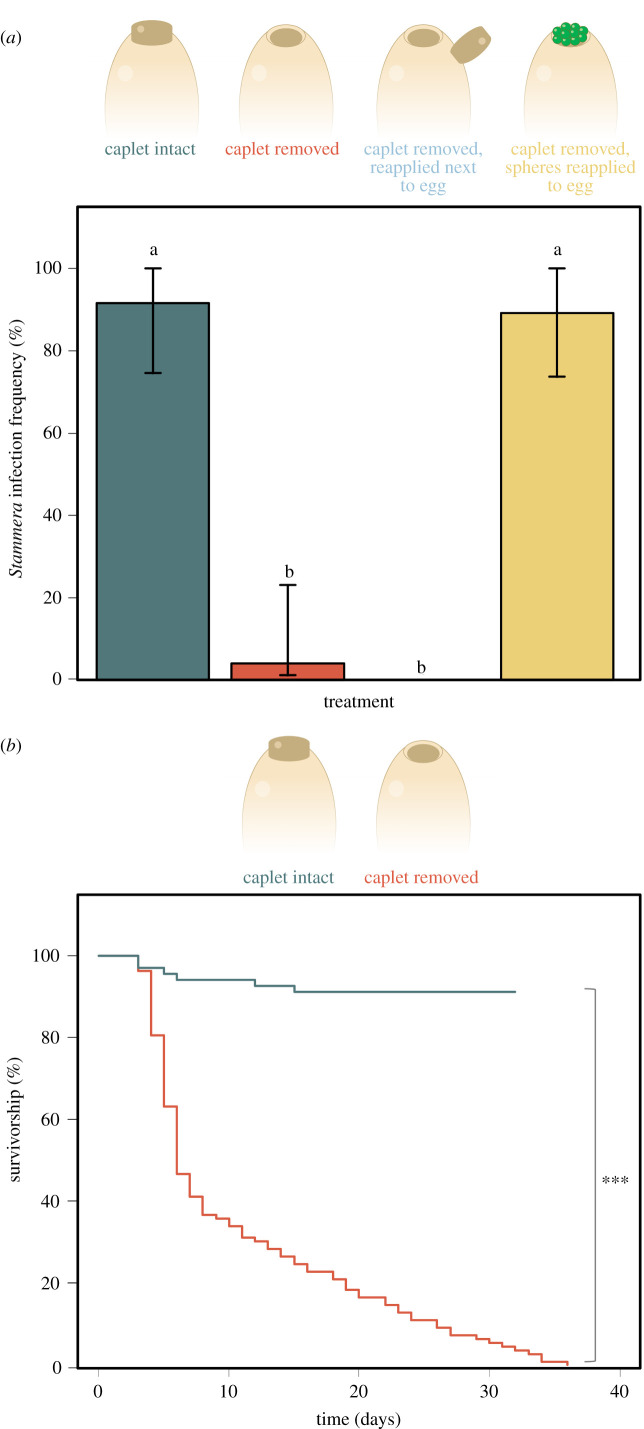
(*a*) *Stammera* infection frequencies in *C. alternans* larvae following experimental manipulation of the egg caplet (Pearson's *χ*^2^-squared test, *χ*^2^ = 68.62, d.f. = 3, *p* < 0.001). Bar coloration signifies the experimental treatment. Number of samples = 87 larvae; Caplet intact (24), Caplet removed (19), Caplet reapplied (23) and Spheres reapplied (21). Whiskers denote the 95% binomial confidence intervals. (*b*) Larval survivorship (to adult eclosion) following experimental removal the egg caplet. Line coloration signifies the experimental treatment. Asterisks indicate significant differences between treatments (Cox's model, *p* < 0.001). (Online version in colour.)

To confirm that *Stammera* is only acquired by its host prior to hatching, we examined symbiont infection dynamics across four experimental treatments (figure 3*a*): (a) untreated control, (b) eggs whose caplets were removed, (c) eggs whose caplets were removed, then deposited immediately adjacent to the egg, and (d) eggs whose caplets were removed, but all the encased spheres were reapplied to the anterior pole of the egg. Contingency tables (2 × 2) and accompanying Pearson's Chi-squared tests were used to evaluate the effect of experimental manipulation of the egg caplet on *Stammera* infection frequencies in *C. alternans* larvae.

Caplet removal disrupts the transmission of *Stammera,* generating aposymbiotic larvae in *C. alternans* (Chisq test, *χ*^2^ = 28.45, d.f. = 1, *p* < 0.001) (figure 3*a*), consistent with prior findings in *Cassida rubiginosa* (Chrysomelidea: Cassidinae) [31]. Reapplying *Stammera*-bearing spheres to eggs whose caplets were experimentally removed restored symbiont infection rates to levels mirroring the untreated control group (Chisq test, *χ*^2^ = 2 × 10*^−^*^31^, d.f. = 1, *p* = 1) (figure 3*a*), confirming that larval access to spheres while in the egg is critical for infection. *Stammera* infection in both groups was consistently high (91.7% (CI = 74–98%), untreated control; 89.17%, spheres reapplied (CI = 71–97%)) save for a few desiccated individuals that failed to transition onto the host plant. By contrast, caplet placement in the immediate vicinity of eggs lacking these structures did not rescue infection in aposymbiotic *C. alternans* (Chisq test, *χ*^2^ = 36.04, d.f. = 1, *p* < 0.001) (figure 3*a*), despite our observation that larvae actively probed the caplets after hatching. This rules out caplet-sharing among siblings and suggests that a specific developmental window governs symbiont acquisition in cassidines—one that precedes eclosion from the egg. Examples of developmental regulation of symbiont acquisition are common in insects and other invertebrates [26,30,40]. Infection competence can vary significantly across juvenile insect stages, as demonstrated in the bean bug *Riptortus pedestris* (Hemiptera: Alydidae) in its symbiosis with *Burkholderia insecticola* [30]. This ensures that symbiont colonization preferentially occurs during a specific nymphal stage [30], where access to the midgut crypts is subsequently constricted following *Burkholderia* passage [41]. By limiting prolonged access of symbiotic organs to potential pathogens and parasites, these strategies may be especially common in insects acquiring extracellular symbionts from the environment or maternal secretions [42].

### (d) *Chelymorpha alternans* is obligately dependent on *Stammera*

As the impact of *Stammera* on host fitness has only been demonstrated in *C. rubiginosa* [31], we aimed to additionally confirm the symbiont's beneficial role in *C. alternans*. The survivorship of symbiotic larvae was compared with aposymbiotic insects hatching from caplet-free eggs (figure 3*b*; electronic supplementary material, table S1). Aposymbiotic larvae exhibited significantly lower survivorship (none reached adulthood) relative to the symbiotic control (Cox's model, *χ*^2^ = 118, d.f. = 1, *p* < 0.001), demonstrating that *C. alternans*, like *C. rubiginosa*, is obligately dependent on *Stammera* for successful development.

### (e) Maternal investments ensuring extracellular symbiont transmission

**Figure 4.**
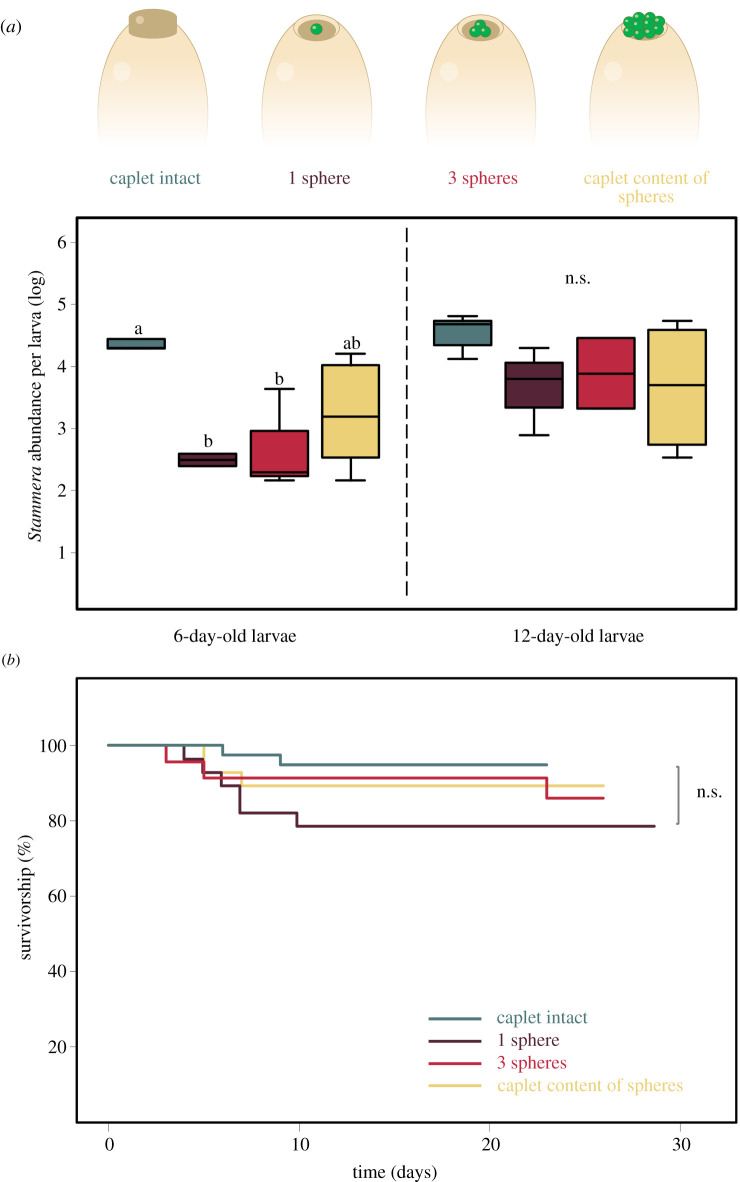
(*a*) *Stammera* relative abundance following the quantification of 16S rRNA gene copy numbers in 6- and 12-day-old larvae. Box coloration signifies the experimental treatment. Lines represent medians, boxes indicate 25–75 percentiles and whiskers denote range. Different letters above boxes indicate significant differences in gene copy numbers (6-day-old larvae: LM, *p* = 0.014; 12-day-old larvae: LM, *p* = 0.27). (*b*) Larval survivorship (to adult eclosion) across treatments. Line coloration signifies the experimental treatment. The asterisks indicate significant differences between treatments (Cox's model, *p* = 0.3). n.s.: not significant. (Online version in colour.)

With more than a third of a caplet's *Stammera* population discarded along with the chorion after hatching (figure 2*a*), we aimed to explore the degree to which female cassidines invest to ensure successful symbiont transmission. Specifically, we asked how many spheres are necessary to initiate infection by *Stammera* and rescue survivorship in aposymbiotic larvae. We addressed this question by capitalizing on the tractability of a transiently aposymbiotic phase during embryo development, along with our ability to individually reintroduce *Stammera*-bearing spheres to eggs whose caplets were experimentally removed (electronic supplementary material, video S1). We monitored symbiont abundance along with larval survivorship across four experimental treatments: (a) eggs whose caplets were left untreated, in addition to caplet-free eggs that were either reinfected with (b) one sphere, (c) three spheres or (d) the entire sphere content harvested from an individual caplet (figure 4).

To verify how infection dosage affects symbiont population density across larval development, we quantified *Stammera* abundance in 6- and 12-day-old insects spanning all four experimental groups. While symbiont density did vary initially across treatments in 6-day-old larvae (LM, *F*_3,11_ = 5.65, *p* = 0.014), these differences were no longer significant when we sampled again 6 days later (LM, *F*_3,11_ = 1.5, *p* = 0.27) (figure 4*a*), indicating that *Stammera*'s population recovered across all groups. Reflecting the consistency in symbiont abundance irrespective of initial inoculation dose, all four treatments reached adulthood at a similar rate (Cox's model, *χ*^2^ = 4, d.f. = 3, *p* = 0.3) (figure 4*b*; electronic supplementary material, table S1), in contrast with the low survivorship recorded for aposymbiotic insects (figure 3*b*). Since our bioassays featured larvae, future studies will assess how variation in symbiont supply shapes the overall fitness of the beetle host. Initial differences in *Stammera* abundance did not impact larval survivorship (figure 4), but potential metabolic costs incurred during that stage may still carry over to pupae and adults, shaping subsequent fitness. As demonstrated in aphids, differences in symbiont density early in insect development are correlated with fitness consequences that extend beyond the survivorship of immature stages, including growth rate and lifetime reproductive output [43]. Quantifying a broader set of fitness parameters in *C. alternans* relative to variation in *Stammera* titres will complement these efforts [43,44], yielding important insights into symbiont density dependence in obligate partnerships.

As a single symbiont-bearing sphere is sufficient to ensure the successful acquisition of *Stammera* by *C. alternans*, our findings indicate that females invest nearly 12 × that amount to mitigate the risk of symbiont loss during transmission. We do note that our experiments were conducted under controlled conditions that may not reflect the environmental challenges encountered by cassidine eggs in the field [36], ranging from sharp fluctuations in temperature and humidity, to high UV exposure on plant leaves. Accounting for the drastic impact of aposymbiosis on host development (figure 3*b*), host investment is likely justified and optimized given the susceptibility of many obligate symbionts to abiotic challenges [45–47]. Our findings are consistent with previous studies examining maternal investments relative to symbiont transmission routes [27,28]. Among plataspid stinkbugs, mothers supply 10 × the number of symbiont cells that is minimally required for the extracellular transfer of *Ishikawaella* [28], possibly also reflecting the prolonged exposure of the symbiont-bearing capsules to environmental challenges during embryo development in the egg [27]. This contrasts with the selective and finely tuned mechanisms ensuring the vertical transmission of intracellular symbionts in insects [19,20,48,49]. In aphids, *Buchnera* cells are released from maternal bacteriocytes, where they are eventually endocytosed by cells fated to become bacteriocytes in the developing embryo [19]. *Buchnera* cells that are not transmitted are ultimately tagged and recycled by the host through Rab7 recruitment and lysosomal activity [50].

By upgrading the nutritional physiology of cassidines through the production of pectinases that function in the gut lumen [31], the extracellular localization of *Stammera* is likely selected for given the complications associated with bacterial enzyme translocation across eukaryotic host membranes [51,52]. While an extracellular placement offers a streamlined path for pectinases to reach the gut, a trade-off may govern how efficiently *Stammera* is packaged and transmitted across host generations. Here, we observe that maternal allocation of *Stammera*-bearing spheres appears to exceed the threshold required for successful symbiont transfer, likely reflecting the abiotic challenges encountered by the caplet while the embryo completes its development in the egg. Our findings indicate that, for a microbe, living outside a cell incurs host investments divergent from those that would be expected from a strictly intracellular lifestyle.

## 3. Methodology

### (a) Insect rearing

A laboratory culture of *Chelymorpha alternans* is continuously maintained at the Max Planck Institute for Biology in Tübingen, Germany. The insects are reared in mesh containers (30 × 30 × 35 cm) along with their host plant *Ipomoea batatas*. Experimental treatments were maintained in climate chambers at a constant temperature of 26°C, humidity of 60% and long light regimes (14.30 h/9.30 h light/dark cycles).

### (b) Scanning electron microscopy

Egg clutches and caplets of *C. alternans* were either air-dried or chemically fixed and critical point-dried, mounted on stubs and sputter-coated with a 10 nm thick layer of platinum (CCU-010, Safematic). Egg clutches were fixed with a solution of 2.5% glutaraldehyde in phosphate-buffered saline (PBS) for 3 days at room temperature, washed and dehydrated in a graded ethanol series with 24 h incubation for each step, followed by critical point drying (CPD300, Leica). Egg caplets were also fixed in 2.5% glutaraldehyde in PBS and post-fixed with 1% osmium tetroxide for 1 h on ice. Ethanol series, critical point drying and sputter-coating were performed as described for the egg clutches. Samples were examined with a field emission scanning electron microscope (Regulus 8230, Hitachi High Technologies) at an accelerating voltage of 3 kV.

### (c) Fluorescence *in situ* hybridization

To localize *Stammera* within the egg caplet, we employed fluorescence *in situ* hydrization (FISH) using semi-thin section preparations. An oligonucleotide probe specifically targeting the 16S rRNA sequence of *Stammera* in *C. alternans*, SAL1055 (5′-GUAGAAGUGCUUUCGAGAACACUA′-3), was designed using ARB software [53]. Eggs were incubated at 26°C and 60% humidity, and sampled at days 7, 9 and 10 after egg oviposition. Eggs were then fixed in 4% formaldehyde (in PBS, v/v) (Electron Microscopy Sciences, PA, USA) at 4°C during 12 h under moderate agitation, and embedded in Paraplast High Melt (Leica, Germany). The paraffin-embedded eggs were cross-sectioned at 10 µm using a microtome and mounted on poly-l-lysine-coated glass slides (Sigma-Aldrich, MO, USA) using a water bath. Egg sections were left to dry in vertical position at room temperature for 4 h and baked at 60°C for 1 h for tissue adherence improvement. They were dewaxed with Roti^®^-Histol (Carl-Roth, Germany) in three consecutive steps for 10 min each followed by decreasing ethanol series of 96, 80, 70 and 50% (v/v) for 10 min each and then washed in milliQ water for 10 min. Slides were dried at 37°C for 30 min and sections were surrounded by a PAP-pen circle (Sigma-Aldrich, MO, USA) to avoid buffer leaking during hybridization. The probe SAL1055 doubly labelled with the fluorophore Cy5 was dissolved at 5 ng µl^−1^ in hybridization buffer containing 35% formamide, 80 mM NaCl, 400 mM Tris-HCl, 0.4% blocking reagent for nucleic acids (Roche, Switzerland), 0.08% SDS (v/v) and 0.08% dextran sulfate (w/v). Fifty microlitres of hybridization buffer was used per egg section. The slides were placed in a hybridization chamber at 46°C for 4 h with KIMTECHScience precision wipes (Kimberly-Clark, TX, USA) partially soaked in 35% formamide to maintain a humid atmosphere. Egg sections were rinsed in pre-warmed 46°C washing buffer (0.07 M NaCl, 0.02 M Tris-×HCl pH 7.8, 5 mM EDTA pH 8 and 0.01% SDS (v/v)) and transferred to fresh pre-warmed washing buffer for 15 min followed by 20 min in 1× PBS, 1 min in milliQ water, and a quick wash in ethanol 96% (v/v), and dried at 37°C for 20 min. All sections were mounted using ProLong^®^ Gold antifade mounting media (Thermo Fisher Scientific, MA, USA) and cured overnight at room temperature. Images were visualized using a dual system Zeiss LSM 780 & Airyscan detector.

### (d) Quantifying symbiont-bearing spheres and *Stammera* relative abundance in the egg caplet

DNA was extracted from *C. alternans* egg caplets using the EZNA^®^ Insect DNA Kit. *Stammera* relative abundance was estimated using an Analytik Jena qTOWER³ cycler. The final reaction volume of 25 µl included the following components: 1 µl of DNA template, 2.5 µl of each primer (10 µM) (electronic supplementary material, table S2), 6.5 µl of autoclaved distilled H_2_O, and 12.5 µl of Qiagen SYBR Green Mix. Primer specificity was verified *in silico* by comparison with reference bacterial sequences in the Ribosomal Database and NCBI. Additionally, PCR products were sequenced to confirm primer specificity *in vitro*. Standard curves (10-fold dilution series from 10^−1^ to 10^−8^ ng µl^−1^) were generated using purified PCR products and measuring their DNA concentration using a NanoDrop TM1000 spectrophotometer. The following cycle parameters were used: 95°C for 10 min, followed by 45 cycles of 95°C for 30 s, 62.7°C for 20 s, and a melting curve analysis was conducted by increasing temperature from 60 to 95°C during 30 s. Based on the standard curve, absolute copy numbers were calculated, which were then used to extrapolate symbiont relative abundance by accounting for the single copy of the 16S gene in *Stammera*'s genome, as previously described [53]. The number of spheres enclosed within each caplet were counted under a stereo microscope (ZEISS Stemi 305) across 20 egg clutches (3 eggs per egg clutch, i.e. 60 caplets).

### (e) Experimental manipulation to elucidate the timing of symbiont acquisition

Egg clutches were collected from three mating pairs to avoid pseudo-replication. The clutches contained 30 or more eggs with well-defined caplets. Eggs were then separated into four experimental treatments: (a) untreated control (24 eggs); (b) eggs whose caplets were removed (19 eggs); (c) eggs whose caplets were removed, then deposited immediately adjacent to the egg (23 eggs); and (d) eggs whose caplets were removed, but all the enclosed spheres were reapplied to the anterior pole of the egg (21 eggs). For treatment (b), caplets were carefully separated from their eggs using sterile dissection scissors and without piercing the developing embryo. Caplet-free eggs were additionally supplied with small ethanol droplets to complete the symbiont-clearing procedure. To test whether *Stammera* can be acquired post-eclosion, dissected caplets were reapplied immediately next to caplet-free eggs using the same procedure as above, resulting in treatment (c). To confirm that *Stammera*-bearing spheres must be accessible to developing embryos prior to hatching for infection to take place, we re-applied the spheres onto the anterior pole of caplet-free eggs, yielding treatment (d).

Three days after hatching, symbiont infection frequencies were validated across all treatments using diagnostic PCR. DNA was extracted from larvae using the EZNA^®^ Insect DNA Kit. PCR-primers targeting the 16S rRNA gene of *Stammera* were used to verify the symbiotic status of the host beetles (electronic supplementary material, table S2). Additionally, specific primers for the *CO1* gene of *C. alternans* were used as a control for the DNA extraction (electronic supplementary material, table S2). Diagnostic PCR was conducted on an Analytik Jena Biometra TAdvanced Thermal Cycler using a final volume of 20 µl containing 1 µl of DNA template, 0.5 µM of each primer and 2× DreamTaq Green PCR Master Mix. The following cycle parameters were used: 5 min at 95°C, followed by 34 cycles of 95°C for 30 s, 57.7 or 62°C (depending on the primer) for 30 s, 72°C for 1 min and a final extension time of 2 min at 72°C.

### (f) Quantifying the impact of symbiont loss on *Chelymorpha alternans*

To assess the impact of symbiont loss on larval survivorship to adulthood, eight egg clutches originating from different *C. alternans* females were collected. Each clutch was then separated into two experimental treatments: (a) untreated control and (b) eggs whose caplets were removed as outlined above. Larvae were observed daily for the assessment of fitness effects across both groups, and survival until adulthood was recorded. All experimental groups were subsampled throughout using *Stammera*-specific diagnostic PCR to confirm the symbiotic status of each treatment.

### (g) Experimental manipulation of symbiont spheres

To estimate the threshold symbiont titre for successful vertical transmission relative to the maternal endowment, we asked how many symbiont-bearing spheres are necessary to ensure the successful propagation of the symbiosis and rescue host fitness. Five egg clutches were collected from separate females and divided into four experimental treatments: (a) untreated control, in addition to caplet-free eggs (as outlined above) that were reinfected with either (b) one sphere, (c) three spheres or (d) the entire sphere content harvested from of a single caplet. Spheres were reintroduced to caplet-free eggs 2 days prior to eclosion through careful deposition on the anterior pole of each egg (electronic supplementary material, video S1).

The impact of the reinfection procedure on *Stammera*'s population was estimated through quantitative PCR. Larvae were sampled from each treatment 6 and 12 days after hatching. DNA was extracted using the EZNA^®^ Insect DNA Kit. *Stammera* relative abundance was estimated using an Analytik Jena qTOWER³ cycler. The final reaction volume of 25 µl included the following components: 1 µl of DNA template, 2.5 µl of each primer (10 µM) (electronic supplementary material table S2), 6.5 µl of autoclaved distilled H_2_O and 12.5 μl of Qiagen SYBR Green Mix. Standard curves (10-fold dilution series from 10^−1^ to 10^−8^ ng µl^−1^) were generated using purified PCR products and measuring their DNA concentration using a NanoDrop TM1000 spectrophotometer. The following cycle parameters were used: 95°C for 10 min, followed by 45 cycles of 95°C for 30 s, 62.7°C for 20 s, and a melting curve analysis was conducted with increasing temperature from 60 to 95°C during 30 s.

To assess the impact of symbiont reinfection on larval survivorship to adulthood, eclosing larvae were observed daily for the assessment of fitness effects across all four groups, and survival until adulthood was recorded as outlined above.

### (h) Statistical analyses

Symbiont population dynamics during egg development were analysed using a general linear model, after reverse transformation and validation of a normal distribution, and using time as a fixed factor. To investigate the number of spheres required to reconstitute symbiont colonization, *Stammera* relative abundance was also analysed using a general linear model, after validation of the normal distribution and using the different treatments as a fixed factor. For these two statistical models, the *post hoc* Tukey HSD test was performed using the ‘glht’ function with Bonferroni corrections. Pearson's *χ*^2^ tests were used to evaluate the effect of experimental manipulation of egg caplets on *Stammera* infection frequencies in *C. alternans* larvae. *Chelymorpha alternans* survival rate until adulthood was analysed across the different experimental treatments with a proportional hazards regression [54] and visualized by computing the Kaplan–Meier survival functions [55]. Statistical analyses were performed in R v. 3.5.3 [56], using the prop.test() function to obtain the 95% confidence intervals for a binomial distribution, the *survival* package for survival analyses [57], *multcomp* package for *post hoc* Tukey test [58] and *GrapheR* package for graphics [59].

## Data Availability

Data and statistical analyses are available from Figshare: https://doi.org/10.6084/m9.figshare.19237194.v1 and in the electronic supplementary material [60].
